# Perinatal Clinical Antecedents of White Matter Microstructural Abnormalities on Diffusion Tensor Imaging in Extremely Preterm Infants

**DOI:** 10.1371/journal.pone.0072974

**Published:** 2013-08-29

**Authors:** Ulana Pogribna, Xintian Yu, Katrina Burson, Yuxiang Zhou, Robert E. Lasky, Ponnada A. Narayana, Nehal A. Parikh

**Affiliations:** 1 Department of Pediatrics, Division of Neonatology, University of Texas Health Science Center, Houston, Texas, United States of America; 2 Department of Diagnostic and Interventional Imaging, University of Texas Health Science Center, Houston, Texas, United States of America; 3 Center for Perinatal Research, The Research Institute at Nationwide Children's Hospital and The Department of Pediatrics, Ohio State University College of Medicine, Columbus, Ohio, United States of America; University of Maryland, College Park, United States of America

## Abstract

**Objective:**

To identify perinatal clinical antecedents of white matter microstructural abnormalities in extremely preterm infants.

**Methods:**

A prospective cohort of extremely preterm infants (N = 86) and healthy term controls (N = 16) underwent diffusion tensor imaging (DTI) at term equivalent age. Region of interest-based measures of white matter microstructure - fractional anisotropy and mean diffusivity - were quantified in seven vulnerable cerebral regions and group differences assessed. In the preterm cohort, multivariable linear regression analyses were conducted to identify independent clinical factors associated with microstructural abnormalities.

**Results:**

Preterm infants had a mean (standard deviation) gestational age of 26.1 (1.7) weeks and birth weight of 824 (182) grams. Compared to term controls, the preterm cohort exhibited widespread microstructural abnormalities in 9 of 14 regional measures. Chorioamnionitis, necrotizing enterocolitis, white matter injury on cranial ultrasound, and increasing duration of mechanical ventilation were adversely correlated with regional microstructure. Conversely, antenatal steroids, female sex, longer duration of caffeine therapy, and greater duration of human milk use were independent favorable factors. White matter injury on cranial ultrasound was associated with a five weeks or greater delayed maturation of the corpus callosum; every additional 10 days of human milk use were associated with a three weeks or greater advanced maturation of the corpus callosum.

**Conclusions:**

Diffusion tensor imaging is sensitive in detecting the widespread cerebral delayed maturation and/or damage increasingly observed in extremely preterm infants. In our cohort, it also aided identification of several previously known or suspected perinatal clinical antecedents of brain injury, aberrant development, and neurodevelopmental impairments.

## Introduction

The prevalence of neurodevelopmental impairments (NDI) may be increasing as a consequence of improved survival in extremely preterm infants (EPI) [1], [2]. Diffuse non-cystic white matter abnormalities may represent the neuropathologic correlates of intellectual, attention, and behavioral impairments that are commonly observed in EPI survivors [3]–[5]. Perinatal risk factors associated with the development of such insults or abnormal brain development have not been thoroughly investigated and remain largely unknown [6]–[8]. Additionally, diagnosis with cranial ultrasound or conventional magnetic resonance imaging (MRI) at term has been challenging because these abnormalities are either not readily visible or less reliably detected with these modalities [9]–[10].

Recent MRI advances that permit quantification of water diffusion and anisotropy *in vivo* using diffusion tensor imaging (DTI) have facilitated sensitive detection of microstructural injury and aberrant brain development in very preterm infants [11]–[13]. DTI produces quantitative measures, including fractional anisotropy (FA) and mean diffusivity (MD) that are sensitive to microstructural white matter abnormalities, thus making it a powerful diagnostic tool and potential early imaging biomarker for NDI in EPI [12], [13]. An in-depth assessment of perinatal antecedents of DTI abnormalities may identify new pathways to injury, brain development, and/or neuroprotection and advance the evidence base for using DTI as a robust imaging biomarker. The goals of our study were two-fold: 1) to elucidate differences in brain development and abnormalities in vulnerable white matter regions using DTI between EPI and healthy term infants and 2) to identify antecedents of white matter microstructural abnormalities at term-equivalent age in a prospective cohort of EPI.

## Methods

### Ethics Statement

Institutional IRB approval was obtained from the University of Texas Health Science Center Houston IRB prior to study initiation. Written informed consent for each infant (preterm and term) was obtained prior to enrollment in the study. The consent was approved by IRB and signed by each subject's parents or guardians prior to enrollment and participation in the study.

### Subject Enrollment

Extremely preterm infants cared for in the Children's Memorial Hermann Hospital NICU between May 2007 and October 2010 were eligible for enrollment after 34 weeks post-menstrual age (PMA). Enrolled infants (N = 86) underwent brain MRI with DTI at 38 weeks PMA or prior to discharge, whichever occurred earlier. A control group of 16 healthy term newborns from the Children's Memorial Hermann Hospital Well Baby Nursery were enrolled between July 2008 and January 2010 to undergo DTI within the first 2 weeks after birth.

To be eligible for the study, preterm infants had to be ≤1000 g birth weight and/or ≤29 weeks gestational age; term infants had to be ≥37 weeks gestational age and with weight appropriate for gestational age. Main exclusion criteria for preterm infants were known congenital CNS anomalies; mechanically ventilated with supplemental oxygen >50%, mean airway pressure >15 cm H_2_O, and high frequency oscillator or requiring sedation (to minimize risks to critically ill infants) at the time of enrollment. Main exclusion criteria for term infants were ≥42 weeks gestation or history of perinatal distress or complications [14]. The same data collection and imaging methods were utilized for both cohorts.

### Imaging and Image Processing

Brain MRI scans were performed on a 3T Philips Achieva scanner, equipped with a 32 channel receiver system and a gradient system capable of producing gradient amplitudes of 80 mT/m with a slew rate of 200 T/m/s. An 8-channel phased array head coil was used for data acquisition. The DTI protocol consisted of a single-shot, spin-echo planar sequence with TR/TE, 6000/61; in plane resolution 1.6×1.6 mm^2^, field of view 180 mm^2^; 112×112 matrix; and 2-mm contiguous slices. 15 directions of diffusion gradients were used with a b value of 800 s/mm^2^; additional image with no diffusion gradient was obtained (b = 0 s/mm^2^).

Prior to scanning, patients were fed, swaddled, and restrained in a MedVac Infant Vacuum Splint (CFI Medical Solutions, Fenton, MI). Noise was reduced using Insta-Puffy Silicone Earplugs (E.A.R. Inc, Boulder, CO) and Natus MiniMuffs (Natus Medical Inc, San Carlos, CA). All scans were completed without sedation and were supervised by an experienced neonatologist and a neonatal research nurse.

**Figure 1:**
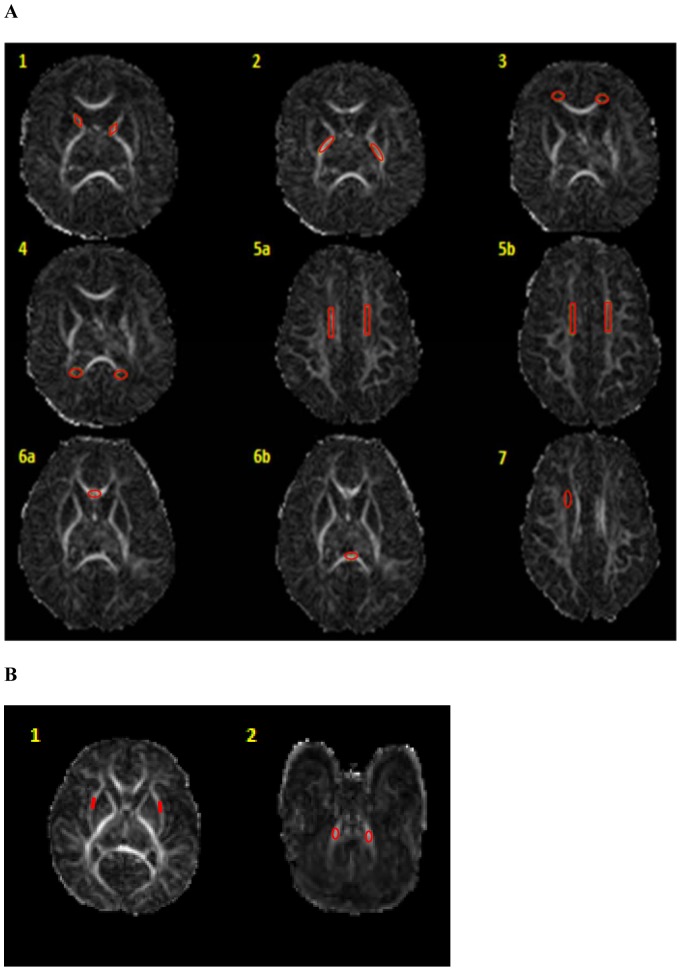
Study and control region of interest templates and placements shown on FA maps. Panel A: 1) Anterior limb of internal capsule, 2) Posterior limb of internal capsule, 3) Frontal periventricular zone, 4) Occipital periventricular zone, 5a–b) Centrum semiovale at two consecutive levels, 6 a–b) Genu and splenium of corpus callosum, and 7) Subventricular zone. Panel B: 1) External capsule, 2) Middle cerebellar peduncles. Same templates were utilized for all scans.

DTI Studio software, Version 3.0.3 (Johns Hopkins University, Baltimore, MD, http://cmrm.med.jhmi.edu) [15] was utilized for analyzing the DTI data. All DTI analyses were performed blinded to the results of cranial ultrasound, anatomical MRI, and clinical variables. Seven vulnerable study and two control regions of interest (ROI) were selected based on prior published data [16], [17]. The seven study white matter ROIs were: anterior and posterior limbs of internal capsule, frontal and occipital periventricular zones, centrum semiovale, genu and splenium of corpus callosum, and the subventricular zone. External capsule and middle cerebellar peduncles were selected as control ROIs. To minimize variability in the ROI placement, ImageJ, Version 1.44p (National Institutes of Health, http://imagej.nih.gov/ij) was utilized for development and placement of ROI templates (one for each ROI) in the native space for fractional anisotropy (FA) and mean diffusivity (MD) measurements (Figure 1). Standardized ROI templates were utilized with manual verification/correction of placement by a trained investigator. The same templates were applied to each DTI scan, minimizing measurement variability between different subjects. All ROI placements were small and centrally placed, minimizing capture of outside non-tract regions. The subventricular zone was only measured in the right hemisphere and the genu and splenium of the corpus callosum were measured centrally. All other regions were measured bilaterally.

### Data Analysis

Forty clinical factors (antenatal, perinatal, and postnatal) were prospectively collected. Clinical variable selection was based on current knowledge, supporting literature, and biological plausibility. Each of the chosen variables had previously been suspected antecedents or previously reported association with early brain injury and/or NDI. All data were entered into a secure database with error checks by qualified neonatal research nurses. All unique identifiers were removed to protect privacy and to blind investigators to clinical history.

**Figure 2:**
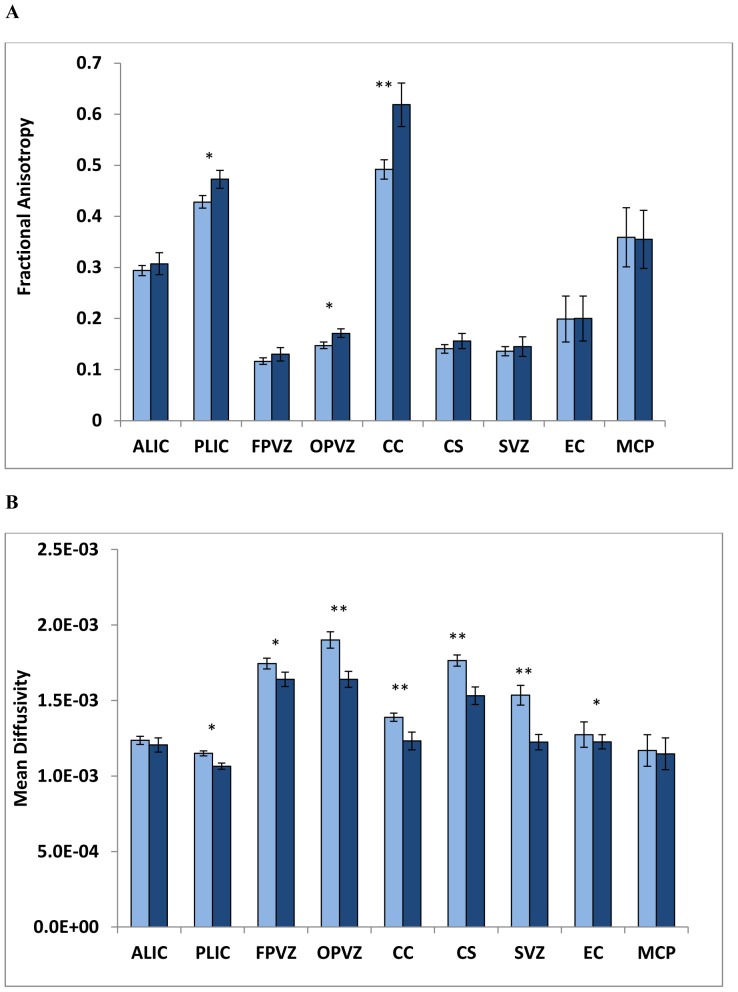
Comparison of mean (SD) fractional anisotropy (A) and mean diffusivity (SD) (B) between extremely preterm infants (light blue) and healthy term infants (navy) for seven study ROIs – ALIC, anterior limb of internal capsule, PLIC, posterior limb of internal capsule, FPVZ, frontal periventricular zone, OPVZ, occipital periventricular zone, CC, corpus callosum (genu and splenium), CS, centrum semiovale, SVZ, subventricular zone; and two control ROIs – EC, external capsule, MCP, middle cerebellar peduncles. *P<0.05 and **P<0.001.

Stata 11/12 IC (Stata Corp, College Station, TX) was used for all data analyses. Two sample t-test, Chi-square and Fisher's exact tests were used as appropriate to describe and compare demographic characteristics among the groups (Table 1). Two sample t-test and Wilcoxon Mann Whitney tests were used to compare FA and MD values between preterm and term infants (Figure 2). Intra-rater reliability for ROI placements was assessed using intra-class correlation coefficients (ICC) (17 cases randomly selected).

**Table 1:** Demographic and clinical characteristics of participating infants.

	Term (n = 15)Mean (SD) or %	EPI (n = 75)Mean (SD) or %
Gestational age at birth, weeks	38.8 (1.0)	26.1 (1.7)**
Birth weight, grams	3177 (381)	824 (182)**
PMA at MRI, weeks	39.1 (1.0)	38.3 (2.1)
Male	53%	4 8%
Maternal age	23.7 (5.3)	28.5 (5.7)*
Private medical insurance	33%	45%
Maternal education: college degree or greater	47%	69%
Maternal hypertension	7%	31%
Maternal insulin-dependent diabetes	0%	5%*
Chorioamnionitis	0%	44%*
Multiple Birth	0%	24%*
Outborn status	0%	9%*
Antenatal steroids given (full course of betamethasone)	N/A	53%
SGA (<10^th^ percentile)	0%	9%*
5 min Apgar score	8.9 (0.3)	6.9 (1.9)**
Delivery room: Intubation at birth	N/A	93%
Delivery room: Resuscitation drug given	N/A	1%
Hypothermia in delivery room	N/A	27%
Hyperthermia in delivery room	N/A	7%
Indomethacin use for PDA treatment	N/A	24%
Surgically treated PDA	N/A	49%
Abnormal head ultrasound (HUS) prior to 28 days of life	N/A	27%
White matter injury on HUS prior to 28 days of life†	N/A	13%
Abnormal conventional MRI at term-equivalent age‡	N/A	61%
Caffeine therapy duration (days)	N/A	51.9 (2.6)
Duration of breast milk use in first month of life (days)	N/A	14.1 (1.0)
NEC	N/A	4%
Duration of mechanical ventilation prior to 36 wks PMA (days)	N/A	19.8 (2.7)

* P<0.05 and.

** P<0.001 in comparison of term and EPI infants.

† Defined as any presence of ventriculomegaly (with or without blood in the ventricles, blood/echodensity or cystic areas in the parenchyma, cystic periventricular leukomalacia, and/or porencephalic cyst evident on cranial US prior to 28 days of life.

‡ Defined as presence of signal abnormalities, brain atrophy, and/or abnormal gray matter or white matter maturation for age.

For the preterm cohort, the selected clinical factors were correlated with FA in bivariate analyses (controlling for PMA at MRI) for each significant ROI. All variables with P<0.25 in bivariate analyses were entered in multiple linear regression analyses with elimination of factors with the highest P values one at a time manually, retaining variables with P<0.1 only [18]. Multiple linear regression models were then developed for each ROI, progressing in chronological order (antenatal, intrapartum, early postnatal, and NICU stay factors). Bootstrapping was used to assess internal validity of developed models. Two-sided P values of <0.05 were considered to indicate statistical significance. No corrections were made for multiple comparisons [18].

## Results

### Demographic Characteristics

75 of the 86 preterm infants and 15 of 16 term infants had high quality DTI scans free of motion artifact and were included in the analyses. Their demographic and clinical characteristics are summarized in Table 1. The preterm and term infants had similar demographic characteristics: gender, private medical insurance, college degree or higher maternal education. Preterm infants were more likely to be born to older mothers, mothers with insulin-dependent diabetes, and chorioamnionitis. Age at MRI was comparable in both groups. Infants excluded due to motion artifacts were similar to the study groups in key demographic and clinical factors.

### Preterm and Term DTI Comparisons

Intra-rater intra-class correlation coefficient for ROI placements was 0.93. FA and MD comparisons between preterm and term infants are shown in Figure 2. As compared to healthy term controls, EPI exhibited significantly lower FA in 3 of 7 and higher MD in 6 of 7 white matter study regions. For control regions, FA and MD values were comparable between EPI and healthy term infants, except for the external capsule region where the mean MD was 4% higher in EPI (P = 0.03).

### Clinical Antecedents

Multiple independent clinical antecedents of FA and MD were identified in the preterm cohort (Table 2). Of antenatal factors, maternal hypertension, outborn status, and chorioamnionitis were adverse factors for abnormalities in FA and MD in the posterior limb of internal capsule and corpus callosum. In clinical terms, presence of chorioamnionitis was associated with MD in the corpus callosum that was 2.0 weeks (95% CI 0.3, 3.8 weeks) less mature than in infants whose mothers did not have chorioamnionitis (based on developmental data from Partridge, et al. [19]) Private medical insurance, a surrogate for better access to prenatal care and higher socioeconomic status, was associated with lower subventricular zone MD. Antenatal steroid use was associated with greater maturation of the subventricular zone (lower MD). No significant intrapartum factors were identified.

**Table 2:** Independent antecedents of fractional anisotropy and mean diffusivity regional abnormalities from the EPI cohort identified in multiple regression analyses.

	Mean FA Difference (95% CI)	Mean MD Difference (95% CI)
**Antenatal**		
Maternal hypertension	PLIC −0.042 (−0.067, −0.017)**	FPVZ 0.060 (−0.011, 0.131)
Private medical insurance	OPVZ 0.012 (−0.001, 0.025)	FPVZ −0.073 (−0.142, −0.005)*
Outborn status		PLIC 0.056 (0.003, 0.108)*
Antenatal steroids		SVZ −0.129 (−0.240, −0.018)*
Chorioamnionitis		CC 0.053 (0.008. 0.098)*
**Intrapartum**		
Rupture of membranes (hours)	PLIC −0.0001 (−0.0002, −0.00001)	OPVZ 0.0003 (−0.00004, 0.001)
Hypothermia		OPVZ 0.101 (−0.014, 0.216)
**Postnatal**		
Birth weight (grams)	CC 0.0001 (−0.00001, 0.0002)	PLIC −0.0001 (−0.0002, −0.00003)**
Caucasian		FPVZ −0.044 (−0.085, −0.003)* CS −0.047 (−0.087, −0.006)*
Female sex		CS −0.060 (−0.127, 0.008)
White matter injury on cranial ultrasound	PLIC −0.041 (−0.075, −0.008)* CC −0.057 (−0.103, −0.011)*	PLIC 0.047 (0.003, 0.092)* SVZ 0.214 (0.046, 0.383)* MCP 0.0001 (0.00004, 0.0002)**
Necrotizing enterocolitis	OPVZ −0.041 (−0.075, −.007)*	OPVZ 0.313 (0.045, 0.580)* CC 0.145 (0.024, 0.266)*
Patent ductus arteriosus	CC −0.031 (−0.065, 0.003)	CC 0.069 (0.022, 0.116)**
Duration of human milk use (per 10 days)	CC 0.037 (0.019, 0.056)†	CC −0.026 (−0.055, 0.003)
Duration of caffeine therapy (per 10 days)		CC −0.012 (−0.023, −0.001)*
Duration of mechanical ventilation prior to 36 weeks PMA (per 10 days)		OPVZ 0.027 (0.001, 0.052)* CS 0.029 (0.012, 0.046)**

* P<0.05,

** P<0.01,

† P<0.001.

(PLIC: posterior limb of internal capsule; FPVZ: frontal periventricular zone; OPVZ: occipital periventricular zone; CC: splenium and genu of corpus callosum; CS: centrum. semiovale; SVZ: subventricular zone; EC: external capsule; MCP: middle cerebellar peduncles).ALIC – anterior limb of internal capsule; PLIC – posterior limb of internal capsule; FPVZ – frontal periventricular zone; OPVZ – occipital periventricular zone; CC – corpus callosum; CS – centrum semiovale; SVZ – subventricular zone; EC – external capsule; MCP – middle cerebellar peduncles.

Several postnatal factors were significant independent adverse antecedents for FA and MD abnormalities in multiple regions (Table 2). White matter injury on cranial ultrasound was adversely associated with FA and MD in several regions, including the MCP, a selected control region. Presence of white matter injury was associated with 4.1 weeks (95% CI 0.8, 7.5) and 5.7 weeks (95% CI 1.1, 10.3) delayed FA maturation in the posterior limb of internal capsule and corpus callosum, respectively [19]. Nine of the ten infants with white matter injury on early cranial ultrasound exam also exhibited an abnormal term MRI scan. Necrotizing enterocolitis (NEC) was associated with injury/delayed maturation of the occipital periventricular zone and corpus callosum. Presence of patent ductus arteriosus (PDA) was a significant adverse antecedent for abnormalities in MD in the corpus callosum. Increasing duration of mechanical ventilation was associated with delayed maturation of occipital periventricular zone and centrum semiovale. Increasing birth weight, female gender, and Caucasian background were favorable factors for multiple brain regions (Table 2). Longer duration of caffeine therapy and human milk use had a favorable effect on corpus callosum microstructural development (Table 2). Increasing duration of human milk use was significantly associated with greater corpus callosum maturation of 3.7 weeks (95% CI 1.9, 5.6) for FA per every 10 additional days of use. Gestational age and birth weight of infants who received human milk were similar to those that did not.

## Discussion

Diffuse non-cystic white matter abnormalities are more commonly encountered in preterm infants than destructive cystic lesions [3], [11]. DTI is a sensitive tool for detecting microstructural white matter abnormalities that may be subtle or appear normal on cranial US. Such abnormalities have been shown to correlate with NDI at 2 years and motor abnormalities at 4 years of age in preterm infants, highlighting DTI's potential as a diagnostic and prognostic tool [20], [21]. Our study adds to the published small number of studied infants comparing microstructural development of EPI to healthy term infants at term-equivalent age [16], [20]–[24]. We observed widespread differences in measures of brain maturation and/or injury as measured by FA and MD in EPI as compared to healthy term controls. The preterm infants exhibited substantial abnormalities in MD in all study regions except anterior limb of internal capsule and in FA in three of the seven regions studied (posterior limb of internal capsule, occipital periventricular zone, and corpus callosum). In two control regions not known to be associated with abnormalities in preterm infants, we found a statistically significant higher MD in EPI in the external capsules. This difference of 4% however, may not be clinically significant.

Investigation of adverse and favorable antecedents for microstructural WM abnormalities is crucial for advancing our understanding of preterm brain injury/development and validating the use of diffusion measures as robust imaging biomarkers. Although important steps have been taken to identify risk factors for white matter abnormalities utilizing DTI, many important antecedents remain unexamined [12], [25]. We investigated 40 clinical variables to provide a more thorough examination of potential adverse and favorable factors. Careful model development through a systematic manual variable selection approach and the use of bootstrapping to confirm internal validity was a major strength of our study. Further strengths include the use of high-field MRI and objective ROI placements for reliable FA and MD measurements. Similar to other observational cohorts however, our study is prone to bias such as confounding by indication and residual confounding. Additionally, our relatively small sample size may have contributed to some false negatives and lack of correction for multiple comparisons likely resulted in a few false positive associations. For example, higher MD in the middle cerebellar peduncles following white matter injury on cranial US may represent a false positive association. As such, larger studies are needed to validate our findings. We also used an ROI based approach that may be better replicated in future investigations using a fully tract-based analysis.

We uncovered several novel antecedents for regional microstructural abnormalities in our extremely preterm cohort. Chorioamnionitis was a strong adverse predictor for abnormalities in the corpus callosum. This association was stronger than previously reported [7]. White matter injury on cranial ultrasound, a known risk factor for brain volume loss and developmental disabilities [26], was also an adverse antecedent in our preterm cohort. Similar to prior reports, we found a correlation between birth weight as well as longer duration of mechanical ventilation and DTI measures [25], [27]. NEC has been associated with white matter injury on cranial ultrasound [28], cerebral palsy [29], and NDI [30]. If validated, our findings of correlation between NEC and adverse occipital periventricular zone and corpus callosum microstructure suggest that NEC associated impairments may be mediated through early injury to these highly vulnerable white matter regions [31].

Several favorable factors were identified in our cohort: maternal private medical insurance, use of antenatal steroids, increasing birth weight, female gender, Caucasian race, increasing duration of caffeine therapy, and longer duration of human milk use. More mature white matter microstructure following increasing duration of human milk use is a novel imaging finding that is consistent with prior evidence of improved cognition in EPI and larger white matter volume in preterm boys [32], [33]. Our findings provide insights into the regional protection possibly afforded by human milk consumption and a quantitative benefit (3–4 week maturation advancement of the corpus callosum per 10 days of human milk use) not described previously. Additional studies are required to comprehensively evaluate the microstructural mechanisms through which maternal milk may enhance cognitive development. A limited amount of information is available about the mechanism of neuroprotection following caffeine in very preterm infants [34], [35]. Our data and those of Doyle et al. [35] indicate that this benefit may be mediated through preserving white matter microstructural integrity.

## Conclusion

Several important and novel postnatal factors are associated with microstructural cerebral injury and/or aberrant development as assessed using DTI. Although many of these factors have also been linked with later NDI, external validation of our results will be important. DTI is emerging as an early imaging biomarker and a sensitive tool to advance our understanding of perinatal brain injury and development. Correlation of DTI measures at term with neurodevelopmental outcomes during early childhood are important next steps.
